# Left Bundle Branch Pacing Improved the Outcome of End‐Stage Hypertrophic Cardiomyopathy: A Case Report

**DOI:** 10.1111/anec.70073

**Published:** 2025-04-04

**Authors:** Manxin Lin, Shufen Huang, Xinyi Huang, Linlin Li, Binni Cai

**Affiliations:** ^1^ Division of Cardiology Xiamen Cardiovascular Hospital, Xiamen University Xiamen Fujian China; ^2^ Division of Echocardiography Xiamen Cardiovascular Hospital, Xiamen University Xiamen Fujian China

**Keywords:** cardiac resynchronization therapy (CRT), heart failure, hypertrophic cardiomyopathy (HCM), left bundle branch block (LBBB), left bundle branch pacing (LBBP)

## Abstract

Patients with hypertrophic cardiomyopathy (HCM) enter the terminal stage when developed left ventricle enlargement and ejection fraction (EF) reduction. The concomitant complete left bundle branch block (LBBB) is considered an important factor related to poor outcome. Previous research suggested that biventricular pacing has limited effects on such patients. We report a case with end‐stage hypertrophic cardiomyopathy who had a miraculous recovery after receiving successful left bundle branch pacing (LBBP).

## Graphical abstract

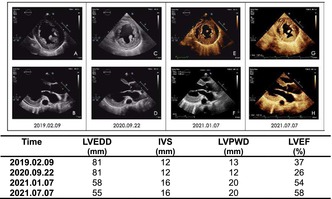

## 1 Introduction

Patients with hypertrophic cardiomyopathy (HCM) enter the terminal stage when they develop left ventricle enlargement and ejection fraction (EF) reduction (Harris et al. 2006). Factors relating to poor outcomes of end‐stage HCM include the existence of left bundle branch block (LBBB), pahologic Q waves, higher levels of New York Heart Association (NYHA) functional classes, and lower left ventricular ejection fraction (LVEF) (Xiao et al. 2015). Complete left bundle branch block (LBBB) is considered an important factor relating to poor outcomes in end‐stage HCM patients, and previous research suggested that conventional biventricular pacing (a form of cardiac resynchronization therapy, CRT) has limited effects on such patients (Pezzulich et al. 2005; Ashrafian et al. 2007; Gu et al. 2017). Left bundle branch pacing (LBBP) is a recently emerged pacing technique that corrects LBBB in order to achieve cardiac resynchronization. Multiple small‐sampled, non‐randomized studies showed that LBBP can result in significant improvements in heart failure patients with LBBB, and is therefore considered a practical and effective method of CRT (Huang et al. 2020; Li et al. 2020; Wu et al. 2021; Guo et al. 2020).

In this article, we report a case of end‐stage HCM with concomitant complete LBBB who received LBBP and had a miraculous recovery marked by significant reversal of cardiac remodeling, LVEF restoration, and NYHA class score improvement. To the best of our knowledge, this is the first report of the application of LBBP in end‐stage HCM patients accompanied by LBBB.

## 2 Clinical Case

**FIGURE 1:**
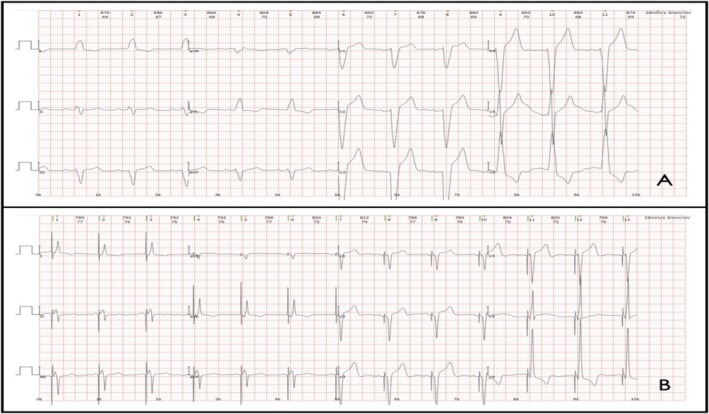
ECGs before and after LBBP. (A) ECG before LBBP: Complete LBBB, QRS 185 ms, Sv1 + Rv5 = 5.6 mV. (B) ECG after LBBP: VAT mode, QRS 105 ms, Sv1 + Rv5 = 7.2 mV.

The patient was a 73‐year‐old male with a history of hypertension and long‐term smoking who denied other significant past medical history or alcohol abuse. He had suffered from shortness of breath upon exertion for over 6 years and was diagnosed with heart failure 2 years ago, with an LVEF of 37%, LVEDD (left ventricular end diastolic diameter) of 81 mm, IVS (interventricular septum) of 12 mm, and LVPWD (left ventricular posterior wall thickness) of 13 mm, while cardiac magnetic resonance imaging (MRI) showed cardiac enlargement and desynchronization. He was then prescribed ARNI, beta‐blockers, spironolactone, and diuretics, but the symptoms worsened gradually. The patient was admitted on September 23rd, 2020, with chief complaints of aggravated shortness of breath accompanied by nocturnal paroxysmal dyspnea and severe edema of lower extremities. Physical examination detected a mild systolic murmur in the apex of the heart and diffused rales in both lungs. CBC, serum biochemistry, ABG, myocardial enzymes, HbA1c, and thyroid hormone levels appeared to be normal upon admission, but the NT‐proBNP level was 3881 pg/mL (reference range < 125 pg/mL). ECG showed sinus rhythm with high voltage of the left ventricle (Sv1 + Rv5 = 5.6 mV) and complete LBBB (QRS 185 ms) (see Figure 1A). Chest X‐ray showed a cardiothoracic ratio of 0.58. Echocardiogram showed an LVEF of 26%, IVS of 12 mm, LVPWD of 12 mm, and LVEDD of 81 mm. Coronary angiography was performed and showed no atherosclerotic stenosis.

After ruling out ischemic, arrhythmogenic, metabolic, alcoholic, Takotsubo, or other secondary cardiomyopathies, genetic testing was performed and revealed four mutation sites, none of which correlate to the HCM clinical phenotype (*SCN5A:NM_198056.3:exon16:c.2773A>G:p.I925V*;*NM_182914.3:c.2646+2TG*; *GAA:NM_000152.5:exon14:c.1935C>A:p.D645E*;*FHOD3:NM_025135.5:exon12:c.1388G>A:p.R463Q*). Family screening showed that one of his sons also had mild left ventricle hypertrophy with an IVS of 12 mm. Combining all clinical, laboratory, and imaging evidence, the patient was eventually diagnosed with hypertrophic cardiomyopathy that had progressed to an end‐stage phase resembling dilated cardiomyopathy, characterized by systolic dysfunction and left ventricular dilation. CRT‐D was indicated, but the patient only consented to CRT‐P due to financial concerns.

**FIGURE 2:**
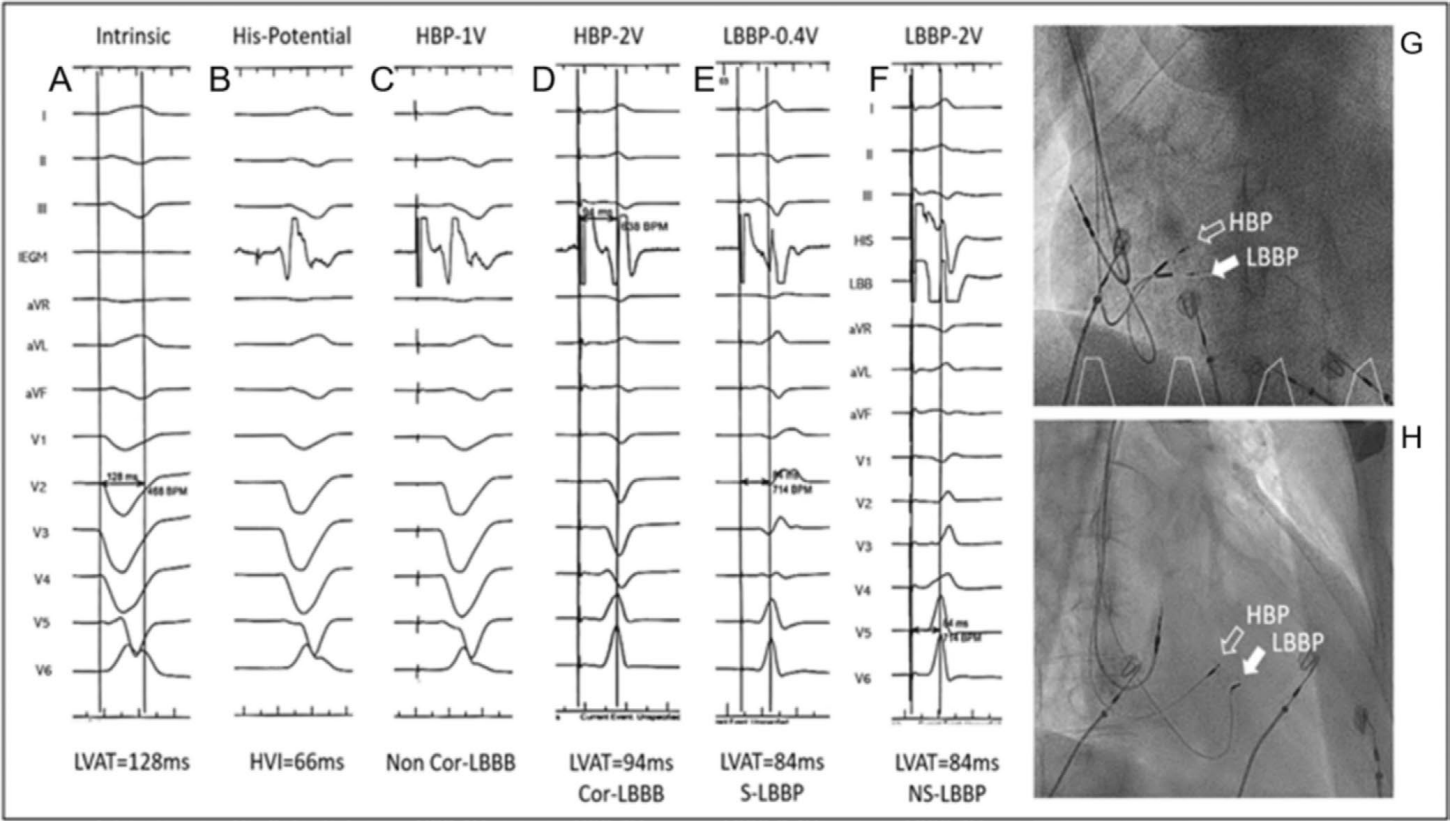
Intracardial electrograms during CRT implantation. (A) Intrinsic rhythm, complete LBBB, LVAT 128 ms. (B) His potential, H‐V interval 66 ms. (C) HBP threshold at 1.0 V/0.5 ms, non‐corrected LBBB. (D) HBP at 2.0 V/0.5 ms, corrected LBBB, LVAT 94 ms. (E) LBBP at 0.4 V/0.5 ms, selective LBBP morphology, LVAT 84 ms. (F) LBBP at 2.0 V/0.5 ms, non‐selective LBBP morphology, LVAT 85 ms. HBP: His bundle pacing; LBBP: Left bundle branch pacing; LBBB: Left bundle branch block; LVAT: Left ventricular activation time.

After careful evaluation and communication, His‐Purkinje conduction system pacing was adopted and a triple‐chamber pacemaker (C2TR01, Medtronic Inc., Minneapolis, MN) was implanted successfully on September 27th, 2020, using techniques proposed by Huang et al. (2019). Two 3830 leads and a C315 Sheath (Medtronic Inc., Minneapolis, MN) were used as the ventricular pacing leads and the delivery catheter, with the 3830 leads fixated on the His bundle area and the left bundle branch area respectively. Another 5076 lead was fixated in the right auricle. His bundle capture and selective left bundle branch capture were confirmed during the procedure (see Figure 2), and the LBBP lead was connected to the RV port while the HBP lead was inserted into the LV port. The pacemaker was set at DDD pacing mode (RA + RV), with PAV/SAV set at 130/100 ms. The post‐operation ECG showed a QRS of 105 ms (see Figure 1B).

**FIGURE 3:**
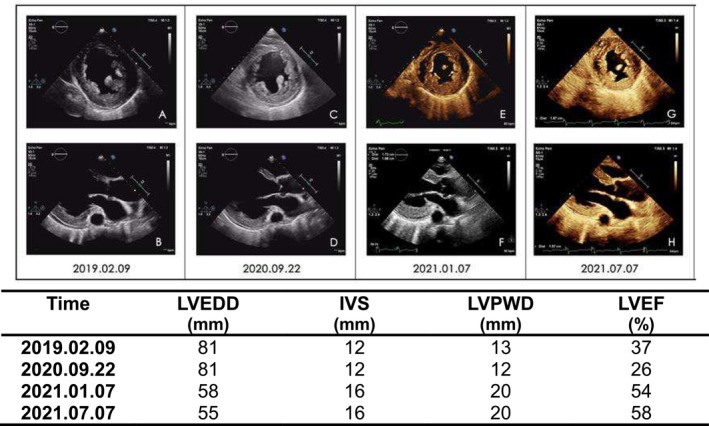
Echocardiograms from left parasternal short‐axis view (A, C, E, G) and apical four‐chamber view (B, D, F, H) showing significant reversal of cardiac remodeling. LVEDD: Left ventricular end‐diastolic diameter; IVS: Interventricular septum; LVPWD: Left ventricular posterior wall thickness; LVEF: Left ventricular ejection fraction.

**FIGURE 4:**
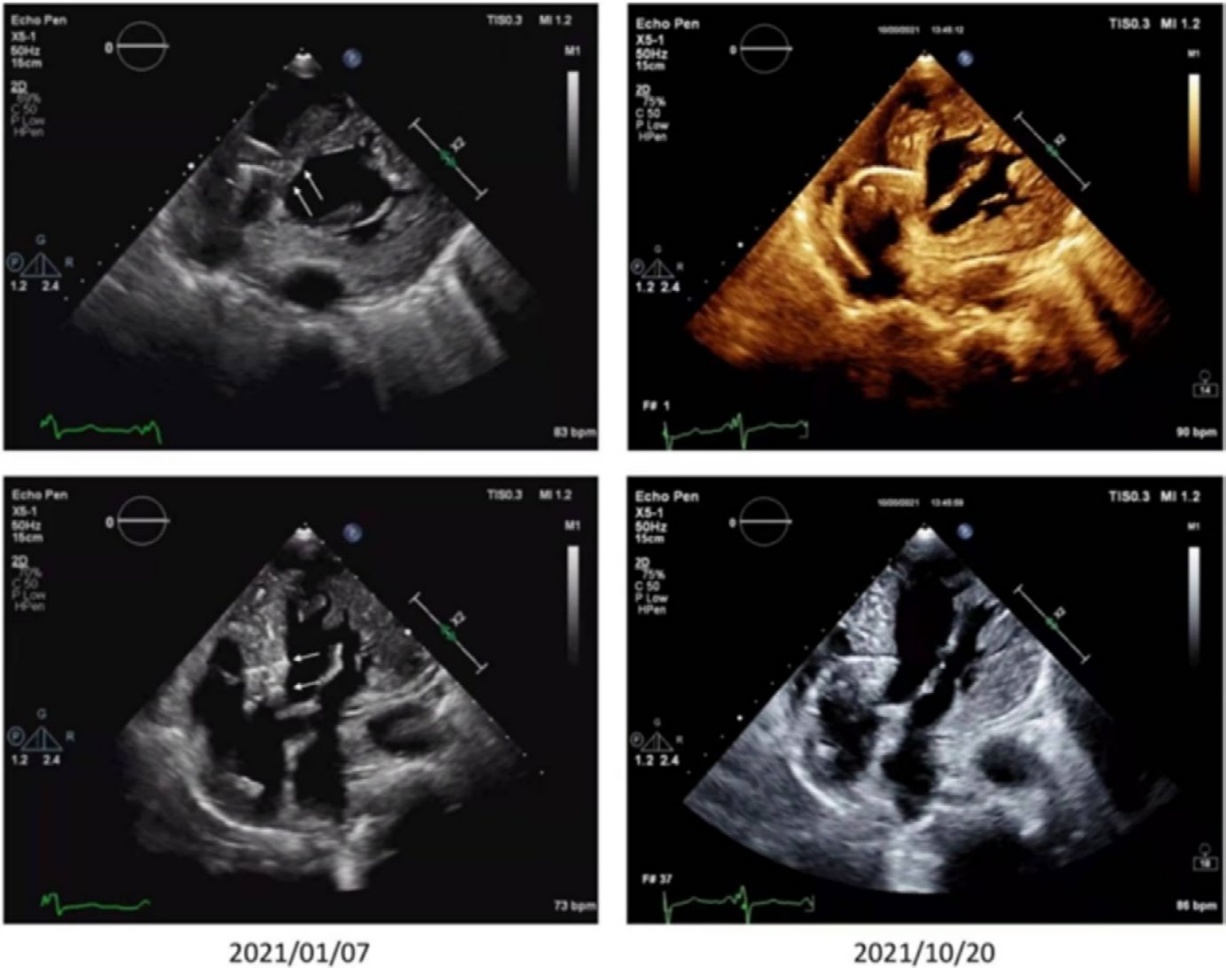
Echocardiograms showing lead positions 3 months and 1 year after CRT implantation. The interventricular septum thickened, but the positions of the leads remained stable.

The patient continued to receive optimal medical therapy. Upon the 3‐month follow‐up, his symptoms improved significantly with normal exercise tolerance. His cardiac function improved to NYHA I, and the echocardiography showed an LVEF of 54%, IVS of 16.1 mm, LVPWD of 19.8 mm, and LVEDD of 58 mm. During the 1‐year follow‐up, the echocardiogram showed significant reversal of cardiac remodeling (see Figure 3). All leads remained intact (see Figure 4), and the LBBP lead parameters including capture threshold (0.5 V/0.4 ms), impedance (390 Ω) and R‐wave amplitude (20 mV) remained stable.

## 3 Discussion

HCM is a common genetic heart disease that is characterized predominantly by left ventricle hypertrophy in the absence of other causes. One infrequent complication of HCM is the development of an end‐stage cardiomyopathic phenotype that occurs in 3%–5% of patients (Killu et al. 2018). These patients develop wall thinning with cavity dilatation, which is usually accompanied by cardiac dysynchrony. In this case, the patient presents with significant left ventricle dilatation and reduced LVEF and only mild wall thickening. In this case, myocardial hypertrophy was masked by the significant dilatation of the heart, which made the diagnosis of HCM seem inappropriate. Yet the IVS and LVPWD increased as the cardiac remodeling reversed as a result of optimal medical therapy and LBBP, confirming the final diagnosis of HCM.

The 2020 AHA/ACC guideline for HCM recommended that patients who develop systolic dysfunction with an LVEF < 50% and LBBB receive CRT treatment (class 2a, level C‐LD) (Ommen et al. 2020). There have been some case reports suggesting patients with end‐stage HCM and concomitant complete LBBB may benefit from biventricular pacing (Pezzulich et al. 2005; Ashrafian et al. 2007), but the cohort study by Gu M. et al. concluded that biventricular pacing has only limited effects on such patients compared to those with dilated cardiomyopathy (DCM) (Gu et al. 2017).

His‐Purkinje conduction system pacing is a recently emerged form of CRT that includes HBP and LBBP and is gaining more and more attention each day. Compared to biventricular pacing, His‐Purkinje conduction system pacing better corrects the cardiac dysynchrony and can achieve shorter QRS. HBP is the most physiological type of pacing, but concerns regarding higher pacing thresholds, lower R‐wave amplitudes, and the potential to develop distal conduction block limited its clinical application. LBBP, as an alternative method, offers lower pacing thresholds and larger R waves and has a lower theoretical risk for development of distal conduction block since it targets the distal conduction system (Huang et al. 2019). Multiple studies have shown that LBBP achieves better electrical synchrony than traditional biventricular pacing and has a better performance in improving echocardiogram data, cardiac functional score, and lead stability (Huang et al. 2020; Li et al. 2020; Wu et al. 2021; Guo et al. 2020).

In this case, both HBP and LBBP were achieved with two 3830 leads, respectively. As was expected, HBP had a higher pacing threshold (2.0 V/0.5 ms) than LBBP when correcting LBBB, which was still acceptable, so we kept the HBP lead and linked it to the LV port as a backup. DDD pacing mode (RA + RV) was adopted, and the AV delay was adjusted to achieve satisfactory cardiac resynchronization. During the follow‐up in the device clinic, the patient exhibited a remarkable response to LBBP with significant reversal of cardiac remodeling, LVEF restoration, and NYHA class score improvement.

Still, further investigation may be indicated. The genetic screening of this patient revealed four mutation sites of undetermined significance, none of which correlate to the HCM clinical phenotype. Whether this patient falls into a special subtype of HCM that responds to LBBP still needs further discussion and investigation.

## 4 Conclusion

We report a case of end‐stage HCM with concomitant complete LBBB who had remarkable reversal of cardiac remodeling and significant ventricular functional improvement after LBBP due to cardiac resynchronization. To the best of our knowledge, this is the first report of the application of LBBP in end‐stage HCM patients with concomitant complete LBBB. The potential clinical benefit of LBBP in patients with end‐stage HCM and LBBB still needs further investigation.

## Author Contributions

**Manxin Lin and Shufen Huang:** collected and analyzed the clinical data and wrote the manuscript. **Xinyi Huang:** helped collect and analyzed the results of echocardiogram. **Linlin Li and Binni Cai:** recruited the subject, helped collect clinical data and reviewed the manuscript. All authors read and approved the final manuscript.

## Consent

Informed consent has been obtained from the patient regarding the use of his clinical data for publication.

## Conflicts of Interest

The authors declare no conflicts of interest.

## Data Availability

The datasets generated during and/or analyzed during the current study are available from the corresponding author, Binni Cai, upon reasonable request.
